# Interacting Effects of Gender and Workplace Conflict on Subjective Social Status and the Mediating Role of Professional Benefits in Registered Nurses: A Cross‐Sectional Study in China

**DOI:** 10.1155/jonm/3317801

**Published:** 2026-05-11

**Authors:** Yu Du, Cheng Liu, Xiao Chen, Yuxia Zhang

**Affiliations:** ^1^ Department of Nursing, Zhongshan Hospital, Fudan University, Shanghai, China, fudan.edu.cn; ^2^ School of Nursing, Fudan University, Shanghai, China, fudan.edu.cn

**Keywords:** gender, nurse, professional benefits, subjective social status, workplace conflict

## Abstract

**Purpose:**

The objectives of this study were to examine whether there is a relationship between workplace conflict and subjective social status and to explore whether gender serves as a moderating variable and perceived professional benefits act as a mediating variable in this relationship.

**Methods:**

Data were derived from a sample of 8575 registered nurses across 29 hospitals in Shanghai. We examined gender differences in the relationship between workplace conflict and nurses’ subjective social status by employing a multilinear regression model that incorporated the interaction between gender and workplace conflict. Additionally, we explored whether professional benefits differently mediated the relationship between workplace conflict and subjective social status for male and female nurses through gender‐specific mediation analyses.

**Results:**

The association between workplace conflict and subjective social status differed by gender (*p* < 0.001). Female nurses who experienced workplace conflict demonstrated a reduced subjective social status (*β* = −0.63, 95% CI: −0.78 to −0.47, *p* < 0.001), with their subjective social status scores further diminishing as the frequency of conflict increased (*β* = −0.65, 95% CI: −0.78 to −0.52, *p* for trend < 0.001). Conversely, among male nurses, a positive correlation was observed (*β* = 0.56, 95% CI: 0.06 to 1.06, *p* for trend = 0.028). Among female nurses, the direct association between workplace conflict and the subjective social status score was statistically significant (*β* = −0.37, 95% CI: −0.49 to −0.25, *p* < 0.001), and an indirect effect mediated through professional benefits was also observed (*β* = −0.43, 95% CI: −0.50 to −0.36, *p* < 0.001). The proportion of mediation was 0.54 (95% CI: 0.45 to 0.64, *p* < 0.001).

**Conclusions:**

Gender plays a significant interactive role in the relationship between workplace conflict and nurses’ subjective social status. Among female nurses, professional benefits function as a partial mediator. Accordingly, it is recommended that healthcare institutions address the concerns and needs of female nurses by enhancing their professional benefits to increase their subjective social status.

**Implications for Nursing Management:**

Managers can improve nurses’ subjective social status by increasing their sense of professional benefits. When encountering workplace conflict, healthcare managers could consider involving male nurses in conflict management and encouraging male nurses who have successfully resolved conflicts to share their experiences in conflict management.

## 1. Introduction

Workplace conflict refers to abuse, threats, and assaults faced by healthcare professionals in the course of their work [1]. Such conflicts encompass not only behaviors that cause physical harm but also verbal abuse, intimidation, and sexual harassment [2]. In hospitals, nurses are the direct providers of care, meaning that they have more interactions with patients and their families, a situation that can lead to high levels of workplace conflict [3]. A meta‐analysis that included 253 studies reported that the prevalence of exposure to any type of workplace conflict was 59.2% among nurses, 56.8% among physicians, and 44.4% among other healthcare professionals [4]. In China, a total of 5 million registered nurses were listed in the 2021 National Health Yearbook [5]. According to a meta‐analysis by Liu [6], the 12‐month incidence of workplace conflict was 71%, meaning that approximately 3.55 million nurses may have experienced various types of workplace conflict, a statistic higher than that reported in other countries. Such conflicts not only cause physical harm but also lead to lasting psychological distress, job burnout, and increased turnover [7–10]. However, most studies in China have relied on self‐reported questionnaires focusing mainly on the frequency of conflict, with little attention to its broader psychological and professional consequences.

Subjective social status (SSS), which is defined as an individual’s perceived place within the social hierarchy [11], influenced by socioeconomic indicators (e.g., occupation and income) as well as social recognition and respect [12]. Low SSS may negatively impact nurses’ professional identity, job satisfaction, and turnover intention [13, 14]. These issues are particularly salient in China, where nurses often face high workloads and relatively low income, further weakening their professional identity and perceived social standing [15–17].

To address the psychological consequences of workplace conflict, researchers have highlighted the importance of positive psychological resources. Perceived professional benefits, defined as nurses’ recognition of the rewards and fulfillment derived from their work, are an important intrinsic motivator [18, 19]. Hobfoll proposed the conservation of resources (COR) theory, which describes how individuals strive to acquire, retain, and protect valuable resources, including personal characteristic resources such as skills and traits that help them resist stress [20, 21]. Based on COR theory, we posit that professional benefits serve as an important mediator between workplace conflict and SSS. Workplace conflict may lead to emotional exhaustion and the depletion of social resources among nurses, thereby weakening their perceived professional benefits and, in turn, reducing their SSS. Accordingly, these intrinsic motivations can also help nurses adjust their professional mindset after experiencing adverse clinical events. When nurses encounter negative events such as workplace conflicts, their sense of professional benefits may be negatively impacted [22], which ultimately affects their perceived SSS. Because professional benefits may offer a new perspective, adopting a benefit‐finding coping approach in the face of adverse events can lead to more effective stress management and contribute to better mental health [23]. Thus, professional benefits may play a mediating role in the relationship between workplace conflict and SSS.

Gender is another factor shaping responses to workplace conflict. When facing workplace conflict, men and women often exhibit different approaches to conflict resolution and feedback. Research shows that women are more likely to adopt cooperative conflict management styles, such as compromise and collaboration, whereas men are more inclined to display assertiveness and confidence in professional settings [24]. In healthcare settings, this mechanism may similarly apply or even be intensified. Since nursing teams are predominantly female, male nurses may possess a “power advantage” when dealing with conflicts, which also serves as a display of “masculine traits” [25]. These different conflict resolution strategies may lead to divergent perceptions of SSS between male and female nurses after experiencing conflict. In gender role theory, sociocultural norms often impose different behavioral expectations on men and women. Women are more often expected to embody roles characterized by “cooperation and caring,” such that their behaviors during conflicts may deviate from these societal expectations. In contrast, the confidence and decisiveness displayed by male nurses during conflicts may align more closely with societal gender expectations for men. Consequently, these differences may lead to divergent psychological responses between male and female nurses after experiencing workplace conflict [26].

**FIGURE 1:**
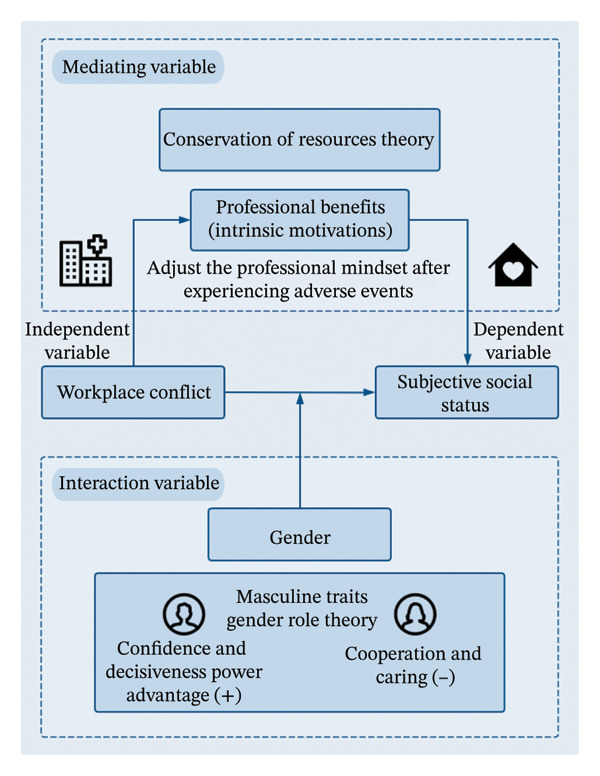
Theoretical model.

Although previous studies regarded gender as a confounding factor, they have failed to take into account the differences in how men and women manage conflicts. Currently, there are no published studies that have examined changes in SSS among male and female nurses following workplace conflict, with gender as a moderating variable. Moreover, incorporating perceived professional benefits as a mediating variable may facilitate the development of interventions to improve nurses’ SSS; however, such research has not yet been reported. Taken together, this discussion leads to the following hypotheses (Figure 1):

• Hypothesis 1: Gender will moderate the influence of workplace conflict on SSS.
• Hypothesis 2: Professional benefits will mediate the relationship between workplace conflict and SSS.

## 2. Methods

### 2.1. Study Design

This was a multicenter, large‐sample cross‐sectional study conducted at 29 hospitals in Shanghai, China. The Strengthening the Reporting of Observational Studies in Epidemiology (STROBE) statement for reporting observational studies was used to report this study [27].

### 2.2. Participants and Procedures

We conducted a survey to assess nurses’ SSS and workplace conflict experiences in Shanghai, one of the most resource‐rich cities in China’s healthcare system. As the city with the greatest number of hospitals across all levels nationwide, Shanghai provides access to a wide range of nursing workplaces across various tiers and categories. Additionally, nurses in Shanghai come from diverse regions across the country, thus contributing to sociocultural heterogeneity that is conducive to exploring the formation mechanisms of SSS. Moreover, with the increasing burden of chronic diseases and an aging population, nurses in Shanghai face increasing work‐related stress, which may highlight their perceptions of role value and workplace conflict.

In 2020, there were approximately 103,100 registered nurses in Shanghai. Considering the reliability and generalizability of the results, we decided to select a sample of 8,000, approximately 7.80% of the registered nurses in Shanghai. In Coleman’s social capital theory, income, educational level, and occupation are the three typical indicators of objective social status and serve as important measures of objective stratification. Objective social status, in turn, influences the perception of SSS. However, our study focuses on examining the social status of nurses as a single occupational group, and income is subject to numerous confounding factors, making it difficult to assess accurately. Therefore, we employed stratified sampling, using educational level as the stratification variable, to categorize nurses into three groups: those with a bachelor’s degree or above, those with an associate’s degree, and those with a technical secondary school diploma or below. By the end of 2020, approximately 11% of all nurses in Shanghai held a technical secondary school diploma or below, 51.24% held an associate’s degree, and 37.76% held a bachelor’s degree or higher. Therefore, our study aimed to select 880 nurses with a technical secondary school diploma or below, 4099 nurses with an associate’s degree, and 3021 nurses with a bachelor’s degree or higher.

We conducted a survey on workplace conflict experiences, SSS, and perceived professional benefits among registered nurses from 29 hospitals using a convenience sampling method. Given the demanding nature of nurses’ work, this approach was chosen to maximize participant convenience and minimize potential discomfort during the data collection process [28]. Eligible participants were included if they met the following criteria: (1) were licensed, practicing clinical nurses; (2) had no cognitive impairments; and (3) provided informed consent. Participants were excluded from the study if they were not registered at the surveyed hospitals or if they were away from Shanghai for further training or on vacation.

### 2.3. Measures

#### 2.3.1. Participant Characteristics and Workplace Conflict Experience

The following characteristics were collected: age, gender, marital status, children, local residence or not, stable housing, degree, and salary.

To improve response rates and completion efficiency, we employed a concise self‐report questionnaire to investigate nurses’ experiences of workplace conflict, with a primary focus on two dimensions—whether they had experienced conflict and the frequency of such experiences. Owing to the dispersed distribution of workplace conflict counts, directly analyzing the raw data may hinder the identification of group differences. However, there is currently a lack of research that clearly defines the grading or rating of conflict frequency. Therefore, during the data analysis, we iteratively refined the regression models and ultimately categorized workplace conflict frequency into three levels: no experience (0 times), low frequency (1–6 times), and high frequency (> 6 times). This classification was based on the distribution characteristics of the sample and the analytical objectives, with the aim being to better compare the effects of different levels of conflict frequency on perceived SSS.

#### 2.3.2. SSS

We used the MacArthur Scale of Subjective Social Status to measure participants’ SSS. The scale was originally developed by Goodman and Adler and has been widely used as a well‐established tool for assessing subjective socioeconomic status [29]. In our study, we used the Chinese version translated by Chen et al. to evaluate nurses’ SSS [30]. The scale divides the social environment and the community environment into ten levels, which are displayed in two ladder‐like structures, with total scores ranging from 2–20. Higher scores indicate elevated SSS. The scale has demonstrated good reliability and validity, with a Cronbach’s *α* for the questionnaire of 0.81 and a test–retest reliability score of 0.86.

#### 2.3.3. Nurses’ Professional Benefits

Nurses’ professional benefits were measured using the Questionnaire of Nurses’ Perceived Professional Benefits, which was designed by Chinese scholars Hu and Liu. Reliability and validity testing were conducted [31]. The questionnaire consisted of 29 items assessing five dimensions of nurses’ perceived professional benefits: (1) positive professional perception (five items); (2) good nurse‒patient relationship (six items); (3) recognition from family and friends (seven items); (4) sense of organizational belonging (six items); and (5) personal growth (five items). Cronbach’s *α* of the questionnaire was 0.958, and the split‐half reliability was 0.938.

### 2.4. Data Collection

We first contacted the nursing department leaders at each study site. After obtaining their support, we recruited a nursing department officer at each site to serve as the investigator. We explained the study’s objectives, target population, and research tools to the investigators and provided standardized training. A pilot survey was conducted at each study site to ensure that all the investigators followed consistent survey procedures. During the pilot survey, we revised certain items to improve clarity and content consistency, thereby enhancing content validity. Once this was confirmed, the investigators administered the questionnaires to eligible nurses at their respective study sites. We facilitated the timely collection and distribution of questionnaires, ensuring that any issues that arose were promptly addressed through direct communication with the investigators. To minimize bias, we did not employ financial incentives or other forms of rewards, as such measures may compromise data accuracy by introducing selection or reporting bias. Furthermore, we did not collect any personally identifiable information, thereby ensuring complete anonymity. This nonintrusive approach was intended to encourage honest and candid feedback.

### 2.5. Data Analysis

Descriptive analyses were conducted to summarize participants’ characteristics and experiences with workplace conflict. Continuous variables are presented as means and standard deviations (SDs), while categorical variables are expressed as percentages. To examine the relationships among participants’ characteristics, experiences of workplace conflict, and SSS, independent *t*‐tests, one‐way analysis of variance (ANOVA), and Pearson/Spearman correlation analyses were performed using SPSS Version 22.0.

Next, to rigorously evaluate the potential moderating effect of gender on the association between workplace conflict and SSS among nurses, we employed multiple linear regression analysis. Specifically, we estimated the regression coefficients (*β*) and corresponding 95% confidence intervals (95% CI) for the interaction term between gender and workplace conflict. Two models were estimated. In Model 1, we did not adjust for any variables, whereas in Model 2, we adjusted for age, marital status, local residence, stable housing, degree, and salary. Concurrently, we performed a trend analysis to formally test the hypothesis of a linear relationship between increasing levels of workplace conflict experience and nurses’ SSS. This analysis calculated the regression coefficient (*β*), its 95% CI, and the associated *p* value for trend (*p* trend) across the ordinal levels or continuous measure of workplace conflict. Statistical significance of the linear trend was assessed using this p‐trend value. These processes were conducted using R statistical software (Version 4.2.3).

Considering that the effect of workplace conflict on outcomes of SSS may rely primarily on professional benefits after experiencing workplace conflict, we separately assessed the indirect effect mediated by professional benefits and the direct effect unmediated by professional benefits in the relationship between workplace conflict and SSS among male and female nurses. The total effect of exposure (workplace conflict) on the outcome (SSS) was divided into the “direct effect” of exposure on the outcome and the “indirect effect” through the mediator (professional benefits). We performed a mediation analysis to calculate the proportion of the “indirect effect” and its 95% CI (simulated by the quasi‐Bayesian Monte Carlo method based on normal approximation) to estimate the proportion of the workplace‐to‐outcome effect attributable to the pathway of professional benefits. This process was conducted using R statistical software (Version 4.2.3).

### 2.6. Ethical Considerations

This study was approved by the Ethics Committee of Zhongshan Hospital, Fudan University (approval number: B2021‐372R) and was performed in accordance with the ethical standards of the 1964 Declaration of Helsinki and its later amendments. Each nurse participating in this study provided informed consent prior to completing the required data collection forms.

## 3. Results

### 3.1. Participant Features

A sample of 8701 questionnaires was investigated across 29 hospitals in Shanghai, resulting in 8575 valid responses and an overall response rate of 98.55%. Among the 8575 registered nurses, the mean (SD) age at baseline was 32.46 (6.26) years; 826 (9.6%) of the participants were male; and 5055 (59.0%) of the participants had experienced workplace conflict. Of those who had experienced workplace conflict, 4507 (52.6%) had experienced 1–6 conflicts, whereas 548 (6.4%) had experienced > 6 conflicts. The SSS score of male nurses was 12.11 ± 4.39, which was significantly higher than that of female nurses (11.05 ± 3.48, *p* < 0.001). The mean (SD) professional benefits score was 115.62 (19.28). Meanwhile, we found significant differences between male and female nurses across different categories of age, marital status, the presence of children, local residence, stable housing, educational degree, annual salary, and workplace conflict (*p* < 0.05). Table 1 presents the descriptive characteristics of nurses in this study, the SSS scores across different characteristics, and the distribution of male and female nurses across these characteristics. Table 2 presents the multivariable analysis of the association between SSS and its influencing factors. Tables 3 and 4 respectively present the correlations among workplace conflict frequency, SSS, and perceived professional benefits in female and male nurses.

**TABLE 1:** Descriptive characteristics of male and female nurses along with their subjective social status scores.

Variable	Male (*n* = 826)	Female (*n* = 7749)	*p* value	Overall (*n* = 8575)	Subjective social status (mean ± SD)	*p* value
Age (years)			< 0.001			0.037
< 30	565 (68.4)	3125 (40.3)		3690 (43.0)	11.12 ± 3.63	
30∼39	199 (24.1)	2920 (37.7)		3119 (36.4)	11.11 ± 3.59	
40∼49	43 (5.2)	1283 (16.6)		1326 (15.5)	11.41 ± 3.40	
≥ 50	19 (2.3)	421 (5.4)		440 (5.1)	10.98 ± 3.59	
Marital status			< 0.001			0.020
Married	276 (33.4)	4571 (59.0)		4847 (56.5)	11.25 ± 3.53	
Unmarried	535 (64.8)	3002 (38.7)		3537 (41.2)	11.04 ± 3.65	
Other	15 (1.8)	176 (2.3)		191 (2.3)	10.88 ± 3.64	
Children			< 0.001			0.755
Yes	176 (21.3)	3981 (51.4)		4157 (48.5)	11.17 ± 3.50	
No	650 (78.7)	3768 (48.6)		4418 (51.5)	11.14 ± 3.67	
Local residence			< 0.001			< 0.001
Yes	488 (59.1)	5277 (68.1)		5765 (67.2)	11.44 ± 3.61	
No	338 (40.9)	2472 (31.9)		2810 (32.8)	10.58 ± 3.48	
Stable housing			< 0.001			< 0.001
Yes	527 (63.8)	5556 (71.7)		6083 (70.9)	11.41 ± 3.56	
No	299 (36.2)	2193 (28.3)		2492 (29.1)	10.55 ± 3.60	
Degree			< 0.001			< 0.001
Below bachelor’s degree	338 (40.9)	5056 (65.2)		5544 (64.7)	10.91 ± 3.72	
Bachelor’s degree and above	488 (59.1)	2693 (34.8)		3031 (35.3)	11.29 ± 3.52	
Initial degree			< 0.001			< 0.001
Below bachelor’s degree	424 (51.3)	6253 (80.7)		6677 (77.9)	10.99 ± 3.53	
Bachelor’s degree and above	402 (48.7)	1496 (19.3)		1898 (22.1)	11.73 ± 3.75	
Salary per year (RMB)			< 0.001			< 0.001
< 100,000	464 (56.2)	2698 (34.8)		3162 (36.9)	10.77 ± 3.87	
100,000–200,000	266 (32.2)	4091 (52.8)		4357 (50.8)	11.11 ± 3.49	
200,000–300,000	72 (8.7)	867 (11.2)		939 (11.0)	12.38 ± 3.13	
> 300,000	24 (2.9)	93 (1.2)		117 (1.4)	13.29 ± 3.59	
Workplace conflict			0.002			< 0.001
Yes	446 (54.0)	4609 (59.5)		5055 (59.0)	10.94 ± 3.48	
No	380 (46.0)	3140 (40.5)		3520 (41.0)	11.47 ± 3.73	
Frequency of workplace conflict (times)			0.008			< 0.001
0	380 (46.0)	3140 (40.5)		3520 (41.0)	11.47 ± 3.73	
1∼6	394 (47.7)	4113 (53.1)		4507 (52.6)	11.03 ± 3.39	
> 6	52 (6.3)	496 (6.4)		548 (6.4)	10.14 ± 4.08	

**TABLE 2:** Multiple linear regression analysis of nurses’ subjective social status.

Variable	*β*	SE	*t*	*p*
Salary	0.123	0.059	10.800	< 0.001
Frequency of workplace conflict	−0.112	0.065	−10.440	< 0.001
Local residence	−0.076	0.098	−5.960	< 0.001
Stable housing	−0.068	0.104	−5.156	< 0.001
Age	−0.035	0.056	−2.564	0.010
Degree	0.029	0.082	2.619	0.009
Marital status	−0.028	0.093	−2.042	0.041

*Note:* *R* ^2^ = 0.043, adjusted *R* ^2^ = 0.042, *F* = 55.05, *p* < 0.001.

**TABLE 3:** Correlation analysis among frequency of workplace conflict, subjective social status, and professional benefits in female nurses.

Variable A Variable B	Frequency of workplace conflict		Professional benefits		Subjective social status	
	*r*	*p* value	*r*	*p* value	*r*	*p* value
Frequency of workplace conflict	—	—	−0.133	< 0.001	−0.100	< 0.001
Professional benefits	−0.133	< 0.001	—	—	0.482	< 0.001
Subjective social status	−0.100	< 0.001	0.482	< 0.001	—	—
Positive professional perception	−0.160	< 0.001	—	—	0.530	< 0.001
Good nurse‒patient relationship	−0.094	< 0.001	—	—	0.404	< 0.001
Recognition from family and friends	−0.112	< 0.001	—	—	0.475	< 0.001
Sense of organizational belonging	−0.117	< 0.001	—	—	0.436	< 0.001
Personal growth	−0.122	< 0.001	—	—	0.433	< 0.001

**TABLE 4:** Correlation analysis among frequency of workplace conflict, subjective social status, and professional benefits in male nurses.

Variable A Variable B	Frequency of workplace conflict		Professional benefits		Subjective social status	
	*r*	*p* value	*r*	*p* value	*r*	*p* value
Frequency of workplace conflict	—	—	−0.086	0.013	0.464	< 0.001
Professional benefits	−0.086	0.013	—	—	0.482	< 0.001
Subjective social status	0.086	0.014	0.482	< 0.001	—	—
Positive professional perception	−0.093	0.008	—	—	0.503	< 0.001
Good nurse‒patient relationship	−0.068	0.051	—	—	0.427	< 0.001
Recognition from family and friends	−0.068	0.050	—	—	0.464	< 0.001
Sense of organizational belonging	−0.094	0.007	—	—	0.445	< 0.001
Personal growth	−0.086	0.013	—	—	0.428	< 0.001

### 3.2. Results of the Interaction

Tables 5 and 6 show the results of multiple regression analyses for the effects of workplace conflict on SSS. Both the crude and adjusted models demonstrate a significant association between workplace conflict and the SSS score. Even when adjusted for 7 SSS‐related factors, the relationship between workplace conflict and the SSS score remained statistically significant (*β* = −0.51, 95% CI: −0.67 to −0.36, *p* < 0.001). In addition, the frequency of workplace conflict was negatively related to SSS (*β* = −0.67, 95% CI: −0.80 to −0.54, *p* for trend < 0.001); as the frequency of workplace conflict increased, nurses were more likely to demonstrate reduced SSS (Table 5).

**TABLE 5:** Interactive effects of gender and workplace conflict on subjective social status (unadjusted model).

Variable	Male			Female			Total		
	*β* (95% CI)	*p* value	*p* for trend	*β* (95% CI)	*p* value	*p* for trend	*β* (95% CI)	*p* value	*p* for trend
Workplace conflict									
No	0			0			0		
Yes	0.51 (−0.09, 1.11)	0.099		−0.63 (−0.78, −0.47)	< 0.001		−0.51 (−0.67, −0.36)	< 0.001	
Frequency of workplace conflict	0.55 (0.05, 1.04)		0.031	−0.65 (−0.78, −0.52)		< 0.001	−0.55 (−0.68, −0.42)		< 0.001
None (0)	0			0			0		
Low (1–6)	0.38 (−0.24, 1.00)	0.227		−0.51 (−0.67, −0.35)	< 0.001		−0.42 (−0.57, −0.26)	< 0.001	
High (> 6)	1.45 (0.18, 2.72)	0.026		−1.62 (−1.95, −1.29)	< 0.001		−1.32 (−1.64, −1.00)	< 0.001	

**TABLE 6:** Interactive effects of gender and workplace conflict on subjective social status (adjusted model).

Variable	Male			Female			Total		
	*β* (95% CI)	*p* value	*p* for trend	*β* (95% CI)	*p* value	*p* for trend	*β* (95% CI)	*p* value	*p* for trend
Workplace conflict									
No	0			0			0		
Yes	0.51 (−0.09, 1.11)	0.097		−0.79 (−0.95, −0.64)	< 0.001		−0.66 (−0.81, −0.51)	< 0.001	
Frequency of workplace conflict	0.56 (0.06, 1.06)		0.028	−0.81 (−0.94, −0.68)		< 0.001	−0.67 (−0.80, −0.54)		< 0.001
None (0)	0			0			0		
Low (1–6)	0.38 (−0.24, 0.99)	0.232		−0.67 (−0.83, −0.51)	< 0.001		−0.56 (−0.71, −0.40)	< 0.001	
High (> 6)	1.58 (0.30, 2.86)	0.016		−1.88 (−2.20, −1.55)	< 0.001		−1.56 (−1.88, −1.24)	< 0.001	

*Note:* This model was adjusted for age, marital status, local residence, stable housing, degree, and salary.

A significant interaction was identified between workplace conflict and gender in relation to SSS (*p* < 0.001). When the data were stratified by gender, distinct patterns became evident. Among female nurses, the frequency of workplace conflict was negatively correlated with SSS (*β* = −0.65, 95% CI: −0.78 to −0.52, *p* for trend < 0.001), which is consistent with the overall findings (Table 6). Conversely, among male nurses, the frequency of workplace conflict was positively related to SSS (*β* = 0.56, 95% CI: 0.06 to 1.06, *p* for trend = 0.028). Moreover, as the frequency of workplace conflict increased, male nurses were more likely to demonstrate elevated SSS (Table 6).

### 3.3. Results of the Mediation

**FIGURE 2:**
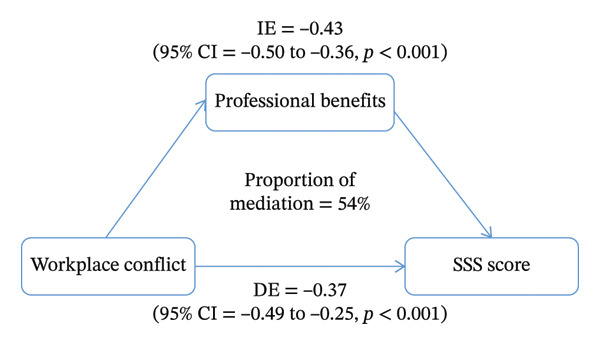
Mediation model of professional benefits among female nurses (*n* = 7749).

The results of the mediation analyses are presented in Table 7 and Figure 2. Among female nurses, the direct association between workplace conflict and the SSS score was statistically significant (*β* = −0.37, 95% CI: −0.49 to −0.25, *p* < 0.001). The mediating effect of the overall association between workplace conflict and SSS mediated by professional benefits was −0.43 (95% CI: −0.50 to −0.36, *p* < 0.001), and the proportion was 0.54 (95% CI: 0.45 to 0.64, *p* < 0.001). In contrast, there was no evidence of mediation through professional benefits among male nurses. The mediating effect of the overall association between workplace conflict and SSS mediated by professional benefits was −0.30 (95% CI: −0.71 to 0.01, *p* = 0.064), and the proportion was −0.55 (95% CI: −6.24 to 4.76, *p* = 0.164).

**TABLE 7:** Decomposition of the total association between workplace conflict and subjective social status into direct and indirect associations mediated by professional benefits.

Exposure: workplace conflict	Mediator: professional benefits (95% CI), *p*	
	Female	Male
Direct effect	−0.37 (−0.49, −0.25), *p* < 0.001	0.86 (0.40, 1.31), *p* < 0.001
Mediation effect	−0.43 (−0.50, −0.36), *p* < 0.001	−0.30 (−0.71, 0.01), *p* = 0.064
Total effect	−0.80 (−0.93, −0.66), *p* < 0.001	0.56 (−0.09, 1.16), *p* = 0.100
Proportion mediated	0.54 (0.45, 0.64), *p* < 0.001	−0.55 (−6.24, 4.76), *p* = 0.164

## 4. Discussion

This is the first study to identify an interaction effect of gender in the relationship between workplace conflict and SSS among nurses. In addition, among female nurses, perceived professional benefits mediate the association between the frequency of workplace conflict and changes in SSS. The results revealed that 59% of the nurses had experienced workplace conflicts, which was consistent with the findings of a meta‐analysis (*n* = 331,544) that estimated the prevalence of workplace conflicts among nurses across 253 studies (61%) [4]. We found that male nurses reported higher SSS scores than female nurses. A possible explanation is that male nurses are more frequently assigned to highly specialized departments, such as intensive care units and emergency departments, where nurses are responsible for rapid decision‐making and high‐level teamwork. These roles are often associated with greater societal recognition and visibility, leading to higher professional identity and external evaluation [32, 33]. Consequently, individuals working in such settings may derive greater satisfaction and a stronger sense of achievement, thereby enhancing their SSS.

After adjusting for age, marital status, local residence, stable housing, education, and salary, we found a significant interaction between gender and workplace conflict in relation to SSS. Female nurses who experienced workplace conflicts reported lower SSS, and their SSS declined further as conflict frequency increased. In contrast, male nurses reported higher SSS under conflict, with SSS rising as conflict frequency increased. This divergent pattern may be related to the gendered dynamics of the nursing profession, which has long been female dominated. As a minority group, male nurses tend to attract more attention—often negative—including being perceived as less caring, facing suspicions of sexual impropriety, or having their masculinity questioned [25, 34]. Consequently, they often feel compelled to demonstrate competence and masculinity through strength, leadership, and technical skills [35]. Workplace conflict provides an opportunity for such displays; compared with women, who are more likely to adopt compromising strategies, men tend to approach conflicts with greater confidence, making them more likely to resolve disputes successfully [24]. Successful conflict resolution may enhance their professional identity and counteract stereotypes, thereby improving their perceived SSS. Moreover, consistent with the “glass escalator” phenomenon [36], men in female‐dominated professions may benefit from implicit advantages that facilitate visibility and recognition, a trend reported in both Western and Asian studies [37–39]. In this study, male nurses were also younger and more highly educated, with stronger adaptability and core competencies. These factors may have enabled them to respond to workplace challenges with confidence, gain growth from such experiences, and further reinforce their perceived SSS.

The incidence of workplace conflicts in healthcare settings is high and difficult to control [40]. However, we found that male nurses who experienced conflicts reported an increase in their SSS, which offers insights for future improvements in conflict management in healthcare, for example, involving male nurses in conflict management strategies. However, considering that the proportion of male nurses is very low or even absent in certain clinical departments and that the underlying mechanisms through which male nurses experience growth in terms of SSS following workplace conflict remain unclear, it is not currently advisable to have male nurses take the lead in conflict management. Nonetheless, it may be valuable to encourage male nurses who have successfully managed conflicts to share their experiences in managing such conflicts, particularly with respect to how they positively adjusted their psychological responses after such events. Therefore, further research is needed to precisely describe the mechanisms by which male nurses express an increase in SSS after experiencing workplace conflicts. In addition, interviews can be conducted with male nurses who have experienced workplace conflict to provide a comprehensive description of their experiences.

The inclusion of professional benefits as a mediating factor provides a more comprehensive understanding of the relationship between workplace conflict and SSS. In our study, the mean professional benefits score was 115.62 (SD = 12.98), which was lower than that reported by Zhou et al. (131.98 [SD = 23.74]) [41]. The reason may be that our study has a larger sample size and includes nurses not only from tertiary general hospitals but also from lower‐level hospitals and specialized hospitals. As a result, nurses in these settings may have relatively limited career development opportunities and a lower sense of professional benefits [42]. This suggests that previous studies on nurses’ sense of professional benefit may not fully represent the current situation of nurses in China, as their sample sizes were relatively small and focused on a single type of hospital. In reality, the professional benefits perceived by nurses might be even lower. Moreover, we found that professional benefits partially mediate the relationship between workplace conflict and SSS. Previous studies have shown that perceived professional benefits can help regulate nurses’ negative emotions, reduce job burnout, which is now common among nurses, and increase nurses’ subjective well‐being [43]. On the basis of positive psychology theory, nurses who feel satisfied with the rewards and benefits their profession brings can promote their overall growth and professional identity [44], which leads to more positive engagement in their work and makes it easier for them to have more positive experiences at work, thereby ultimately providing higher‐quality care to their patients. Such nurses are often less affected by adverse medical events and, even when faced with such events, they are able to cope positively with the event and maintain their perceived SSS [45].

The complexity of the nursing work environment generally leads to a lower perceived social status among nurses, and for female nurses, workplace conflicts further diminish their sense of social status. However, the mediating role of professional benefits offers a new perspective. Compared with the challenging task of changing the clinical environment, providing nurses with more career development opportunities and enabling them to gain the well‐being that the nursing profession can bring may have greater practical significance in clinical settings. For example, in China, where the population is large and aging is a significant concern, timely access to treatment and medical resources remains relatively challenging [46]. However, nurses often have the advantage of easier access to healthcare services, not only for themselves but also for their family members and relatives, who are more likely to benefit from high‐quality medical care. Continued professional development (CPD) keeps nurses’ skills and knowledge current, thereby enhancing the professional performance of nursing and bringing positive benefits to patients, organizations, and individual nurses [47]. This increases nurses’ sense of professional benefit. The research indicates that nurses are motivated to participate in CPD and believe that it benefits both patient care and career advancement, but these programs must be captivating and easily accessible; they must provide a sufficient amount of study time, and the participants’ efforts must be recognized [48]. Research has shown that the greater the degree of organizational support in the work environment is, the greater the degree of nurses’ perceived professional benefits [41, 49]. Additionally, studies have shown that cognitive‒behavioral therapy facilitates nurses’ development of new models of thinking and behavior [50]. Both CPD and cognitive–behavioral therapy can be considered by managers as effective strategies to enhance nurses’ perceived professional benefits.

### 4.1. Limitations

Our study surveyed only registered nurses in Shanghai, so the results may not be generalizable. However, this is a multicenter, large‐sample study, and Shanghai is the leading city in healthcare and nursing around the country; therefore, our results can be representative at the national level. This is a cross‐sectional study rather than a longitudinal design, so it cannot explore causal relationships between variables. However, since workplace conflict was assessed based on experiences across the entire career, while SSS and perceived professional benefits reflect more recent perceptions, there is a temporal sequence between them, which allows for a rough inference of causal relationships. Additionally, owing to the absence of a standardized scale for assessing workplace conflict among nurses, we employed a self‐report questionnaire to investigate the current state of workplace conflict. In the future, the development of a relevant standardized instrument should be considered. Finally, in this study sample, the substantial gender imbalance between male and female nurses may have limited the statistical power of gender comparisons. However, when analyzing and comparing the two groups, we strictly controlled for model fit indices and CIs to ensure the reliability of the results to as great a degree as possible. Future studies may consider the use of quota sampling or other balanced design approaches to further enhance the statistical power and interpretability of gender‐based comparisons.

## 5. Conclusions

To our knowledge, our research is the first to reveal differences in SSS perceptions between male and female nurses after experiencing workplace conflicts. Among male nurses, SSS increased as the frequency of workplace conflict increased, whereas among female nurses, SSS decreased as the frequency increased. Additionally, among female nurses, professional benefits partially mediate the relationship between workplace conflict experience and SSS. Therefore, healthcare managers should consider involving male nurses in conflict management strategies and actively implementing measures to increase nurses’ sense of professional benefits.

## 6. Implications for Nursing Management

This study provides an in‐depth exploration of the relationship between workplace conflict and SSS, introducing gender as a moderating variable and perceived professional benefits as a mediating variable. The findings offer valuable insights for improving the SSS of nurses in China, provide a novel theoretical advancement in the field of occupational health nursing, and offer new practical implications for future interventions. We found that workplace conflict occurs relatively frequently among nurses and has an impact on their perceived SSS. In China, nurses generally report a low sense of social status [15], which negatively affects their work enthusiasm and contributes to undesirable outcomes such as increased turnover intentions. Therefore, nursing managers should pay special attention to changes in nurses’ SSS following workplace conflicts, as well as the role of professional benefits associated with these changes. Given that we found that male nurses reported an increase in their SSS after experiencing workplace conflicts, it is suggested that nursing managers encourage male nurses who have successfully managed conflict to share their experiences, particularly with respect to how they positively adjusted their psychological responses after such events.

Additionally, our findings revealed that among female nurses, professional benefits serve as a mediating factor between workplace conflict and SSS. Enhancing nurses’ sense of professional benefits can improve their perceptions of their SSS. Compared with the challenges of mitigating workplace conflict and occupational stress, enabling nurses to experience the benefits and fulfillment brought by their profession holds greater practical significance. Therefore, nursing managers should provide nurses with more opportunities for professional development, allowing them to experience the well‐being associated with the nursing profession. Then, nursing managers could implement appropriate measures to provide a supportive organizational culture to ensure long‐term support for nurses’ continuous professional development, thereby improving their sense of professional benefits. Furthermore, nursing managers could consider offering more lectures and establishing psychological counseling services for healthcare personnel to help nurses identify irrational negative thoughts, restructure their thinking patterns, and teach them stress management and emotional regulation techniques as doing so would increase nurses’ positive behaviors and enhance their job satisfaction.

## Author Contributions

Yu Du and Cheng Liu contributed equally to this study and are considered cofirst authors.

## Funding

This study is supported by the Shanghai Philosophy and Social Science Program under Award Number 2020BGL018.

## Disclosure

The funder had no role in the design of the study, the collection or analysis of data, or the decision to publish.

## Ethics Statement

This study was approved by the Ethical Committee of Zhongshan Hospital, Fudan University (approval number: B2021‐372R).

## Conflicts of Interest

The authors declare no conflicts of interest.

## Data Availability

The data that support the findings of this study are available from the corresponding author upon reasonable request.
